# Assessment of selected heavy metals in honey samples using flame atomic absorption spectroscopy (FAAS), Ethiopia

**DOI:** 10.1186/s13065-022-00878-y

**Published:** 2022-11-05

**Authors:** Dessie Tibebe, Mohammed Hussen, Marye Mulugeta, Dereje yenealem, Zerubabile Moges, Mohammed Gedefaw, Yezbie Kassa

**Affiliations:** 1 Department of Chemistry, College of Natural and Computational Sciences, University of Gondar, Gondar, Ethiopia; 2 Department of Biology, College of Natural and Computational Sciences, University of Gondar, Gondar, Ethiopia; 3 Department of Natural Resource Management, University of Gondar, P.O. Box: 196, Gondar, Ethiopia

**Keywords:** Honey, Metals, Bees, FAAS, Ethiopia

## Abstract

Honey is a natural, sugary and sticky liquid that is produced from the nectars of flowers by the bees. This study aimed to analyze the concentration of some selected heavy Metals in honey samples. 1 g of honey sample was digested by a hot plate using 9ml of HNO_3_ and 3ml of H_2_O_2_. The concentrations of the heavy metals in the digested were detected using a flame atomic absorption spectrometer. The results of this study found that the concentrations of the heavy metals in the honey samples were ranged from 1.97 to 2.04 µg/g for Zn, 1.93 µg/g to 2 µg/g for Cu, 0.83 to 1.01 µg/g for Mn, 0.25 to 0.45 µg/g for Cr, 0.025–0.031 µg/g for Cd. However, Pb was not detected in all honey samples. Hence, the levels of heavy metals found were below the permitted levels set by the World Health Organization. From the results, the levels of heavy metals found were below the permitted levels set by the World Health Organization. Thus, the heavy metals in the sampled honey are safe for human consumption in these selected areas.

## Introduction

Honey is a natural, sugary and sticky liquid that produces from the nectars of vegetation and/or plants. Honey is one of the most widely required products due to its unique nutritional and medicinal properties [1–3]. Honey bees gather the material, transform by combining with particular substances in their own, deposit, dehydrate, save and leave within the honeycomb to grow up and mature. Extracted honey is a gelatinous liquid foodstuff containing a number of nutritiously important complementary elements [4–8] such as carbohydrates, maltose, sucrose, fructose, glucose, traces metals, organic and inorganic substances and water [9, 10]. High concentrations of these trace toxic elements in honey may result minimized quality set by regular control of food [11–14]. In ancient time the golden yellow liquid honey processed for its medicinal properties [15]. The heavy metals found in the environment can deposit at the hairy bodies of bees hive, flower, herb and water [16]. The toxic metals in the human body, causing the side effects, so honey quality specific elemental content becomes the important issue for human nutrition and safety [17, 18]. High accumulation of toxic heavy metals in plants is hazardous for the food chain and may result in damages to human and animals heaths [19]. Contaminated water and agricultural fertilizers are some major cause of heavy metal contamination in the plant tissues [20]. The presence of Lead, Cadmium and Chromium in bee honey is an evidence of micro polluting metals in the environment [19].

The quality and composition of honey is important to determine its suitability for processing and to meet the demand of the market [21, 22]. Trace amounts of metals Zn, Cu, and Mn are common in honey and not harmful to our health. But, toxic metals Cd, Cr, and Pb possibly will be dangerous to our health in trace or large amount [5–23]. The concentration of heavy metals found above the permitted levels by pollution standards are a threat to human health [24]. The novelty of this study is that there is no metal contaminant investigation done in South Wollo Zone. Environmental Protection Agency listed Cr as one of the 129 priority pollutants and most noxious heavy metals [25]. Therefore, the aim of this study was to determine the concentration of heavy metals in honey samples and standard quality levels from various parts of South Wollo.

## Materials and methods

### Study area description

**Fig. 1:**
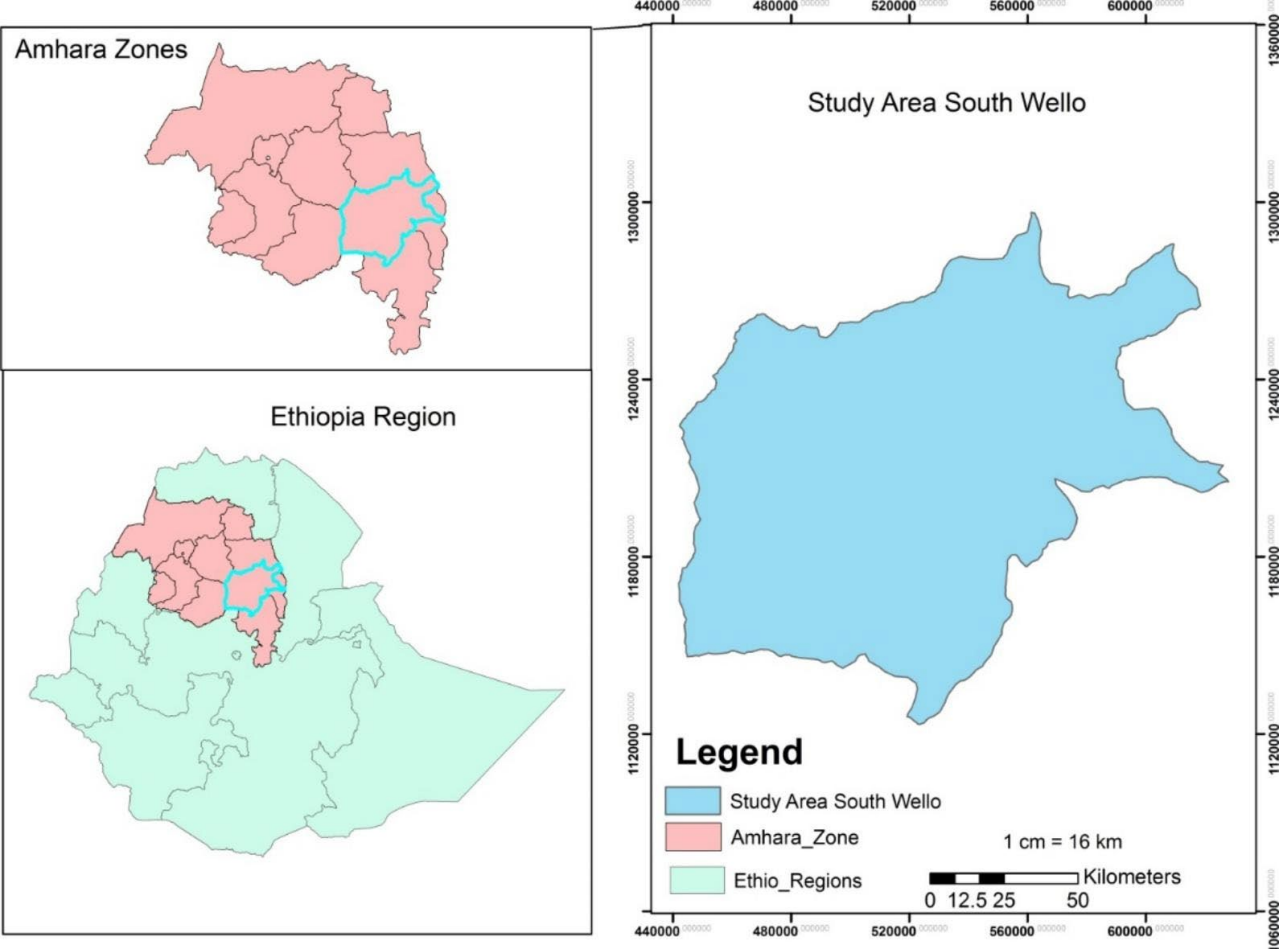
Location map of the study area

This study is conducted in South Wollo Zone in Amhara Regional state of Ethiopia. From 23 woredas, six highly productive Woredas were selected. The selected woredas are Tenta, Mekdela, Legambo, Dessie Zuria, Kalu and Tehuledere (Fig. 1).

### Sample collection

Samples were collected from six districts of South Wollo Zone of Amhara regional state. From this Zone, six different types of honey samples were collected. Four samples were taken from the woreda town market in each woreda selected by cluster sampling technique and composited into one sample to represent the woreda honey sample. The honey samples were stored in plastic jars and temporarily kept at Adjara preparatory and high school laboratory in a refrigerator until it was transported to the Gondar University laboratory and kept there in the refrigerator until analysis.

### Analytical procedures to heavy metal analysis

Honey samples were heated at 65^o^c on a water bath until liquefy to allow easier handling and have more uniform distribution [5–26]. The samples were then cool and weigh for the next analysis. 12 mL of an acid mixture (3:1 ratio of HNO_3_ and H_2_O_2_) were added in 100 ml conical flask containing 1 g of honey. The flasks were heated on the hot plate until the manufacturing of red NO_2_ gases ceases. The content was evaporated, the quantity became decrease to about 3–5 ml but not too dry. After cooling the flask 10 ml of distilled water was added and the solution filtered through the Watmen Number 1 filter paper and transfers to a 25 ml flask. It was then made up to fill with deionized water. Next to this 10 mg/L intermediate standard solution is used to prepare the working solution of the metals (Pb, Cd, Zn, Cu, Mn, and Cr) from 1000 mg/L stock solution and to dilute with 50ml distilled water. Finally, the metals have been determined by atomic absorption spectrophotometer (AAS). Each standard concentration and absorption became used to prepare a curve. The metal concentrations were calculated from the standard curve [6, 27].

### Apparatus and equipment

The laboratory devices that were used for this study include: digital Analytical Balance (Model CTG1200-1200), refrigerator (Model No.LR.1602 England), hot plate, water bath, volumetric flasks, beakers, measuring cylinders, spatula, funnel, filter papers, pipettes and micropipettes, round bottom flask and Atomic Absorption Spectrometer (BUCK SCIENTIFIC MODEL 210VGP).

### Chemicals and reagents

Reagents that were used in the analysis were HNO_3,_ HCIO_4_ and H_2_O_2_ were used for decomopstion of honey samples. And also used during optimization of honey samples. Stock standard solutions (1000 mg/L) containing the metals Pb, Zn, Cu, Cd, Mn, and Cr metals were used for the preparation of calibration standards and in the spiking experiments. Deionized water was used all over the experiment for sample preparation, dilution and cleaning apparatus.

### Calibration of the instrument

The instrument was calibrated using six-seven series of working standards. The working standard solutions of each metal were prepared by diluting the intermediate standard solution. The working standard, the intimidate standard and the value of the correlation coefficient of the Calibration for each heavy metal was shown in (Table 1).

**Table 1:** The intermediate standard solutions used to prepare working standards and correlation coefficients

No	Metal	Intermediate Standards (mg/L)	Working standards (mg/L)	Correlation coefficient of calibration curves
1	Cu	10	0.02,0.5,1,1.5,2,2.5,3	R^2^ = 0.996
2	Zn	10	0.02,0.5,1,1.5,2,2.5,3	R^2^ = 0.991
3	Pb	10	0.01,0.2,0.4,0.6.0.8.1	R^2^ = 0.993
4	Cd	10	0.001,0.2,0.4,0.6,0.8,1	R^2^ = 0.996
5	Mn	10	0.02,0.5,1,1.5,2,2.5,3	R^2^ = 0.996
6	Cr	10	0.01,0.5,1,1.5,2,2.5,3	R^2^ = 0.992

### Data analysis

The experiments were conducted in triplicate. Data were calculated as mean± standard deviation (SD) and were performed using SPSS version 20. Some tests such as one-way ANOVA were used for data analyzing.

## Results

From the results obtained, the concentration of heavy metals varied from one sample to another depending on climatic conditions, botanical origin, storage techniques and extraction methods. As shown in the (Tables 2 and 3).

**Table 2:** Concentration of heavy metals in honey samples in µg/g (n = 3)

Sample areas	Statics	Zn	Cu	Mn	Cd	Cr	Pb	
Tenta	± SD	2.01 ± 0.01	1.92 ± 0.0	0.83 ± 0.0	0.03 ± 0.0	0.25 ± 0.0	ND	
	RSD%	0.49	1	1.2	11	8		
Mekdela	± SD	1.98 ± 0.1	1.93 ± 0.0	1.01± 0.0	0.03 ± 0.0	0.28 ± 0.0	ND	
	RSD%	2.5	2	0.9	4	10		
Legambo	± SD	2.04 ± 0.1	1.96 ± 0.0	1 ± 0.0	0.03 ± 0.0	0.32 ± 0.0	ND	
	RSD%	2.9	1	1	7.1	12		
Tehuledere	± SD	1.98 ± 0.0	1.99 ± 0.0	0.93 ± 0.0	0.03 ± 0.0	0.35 ± 0.0	ND	
	RSD%	1	0.5	1	3.7	8.5		
DessieZuria	± SD	2.04 ± 0.0	2.01 ± 0.0	0.85 ± 0.0	0.03 ± 0.0	0.33 ± 0.02	ND	
	RSD%	0.49	0.5	1.2	6.8	6		
Kalu	± SD	1.97 ± 0.1	2 ± 0.0	0.94 ± 0.1	0.031 ± 0.0	0.45 ± 0.0	ND	
	RSD%	2	1	6.4	9.7	2.2		

ND: not Detected

**Table 3:** The concentrations of metals in different studies and WHO standards in (µg/g)

Studies	Zn	Cu	Pb	Cd	Mn	Cr	Reference
WHO (2002)	350	300	0.5	0.5	5.5	100	[40]
Iran(by Razzagh and et al., 2015)	2.03–6.8	-	0.08–0.12	-	-	-	[15]
Ethiopia (by Gebru and et al., 2015)	0.65–0.93	0.13–0.23	0.03–0.1	0.02–0.03	-	-	[36]
Kenea (Erene 2012)	1.01-2,1	0.07–0.24	0.01–0.05	0.01–0.05	-	-	[5]
Ethiopia (Shibru 2014)	-	0.085–0.133	0.152–0.201	0.07–0.222		-	[33]
Nigerea(EzehErnesti and et al., 2018)	-	-	0.175	0.088	-	6.67	[34]
Ethiopia (Wolde and et al., 2018)	1.92–4.22	ND-0.468	ND	ND-0.69	ND-0.885	1.20–4.33	[37]
Ethiopia (Ashenafi 2018)	0.062–0.33	0.027–0.0697	-	-	0.0693–0.815	-	[14]
Turky(Tuba and et al., 2015)	0.48	0.15	-	-	0.187	-	[25]
Ethiopia 2018 study (present study)	1.97–2.04	1.92-2	ND	0.025–0.031	0.83–1.01	0.25–0.45	

### Method validation

The results of method validation in Table 4 showed that precision, limit of detection, recovery and calibration of instruments are acceptable. The results of precision can be expressed by standard deviation, it was reliable and below P = 0.05, percentage of recovery becomes on the rage (80–120%) [28] and correlation coefficient also above 0.99. The results of analysis of heavy metals are greater than limit of detections. Therefore, the obtained results of heavy metals in honey by this method are accepted and reasonable.

**Table 4:** The method validity

Metal	Sd	LOD	Recovery (%)	Correlation coefficient
Zn Cu Mn Cd Pb Cr	0.01–0.06 0.01–0.04 0.01–0.01 0.001–0.003 ND 0.01–0.04	0.002 0.005 0.005 0.005 - 0.005	80% ± 1 110% s ± 0.5 90% ± 1.26 95% ± 2.5 - 80% ± 2	R^2^ = 0.996 R^2^ = 0.991 R^2^ = 0.993 R^2^ = 0.996 - R^2^ = 0.992

### Validation of the decomposition procedures

To test the effectiveness of the established optimized decomposition; spiking experiments were done. Known amount of each metal 500 µL of Zn, 500µL of Cu, 500 µL of Mn, 250 µL Cd and 250 µL Cr) were spiked from 10 mg/L intermediate standard solution in a round bottom flask containing 1 g of honey sample for each filled with distilled water in a 25 ml marked conical flask then, the spiked samples were digested the same as the developed decomposition method for honey. Each sample was analyzed for one-to-one spiked metals by FAAS. The results of percentage recoveries for the studied metals in honey were between 80 and 110% as indicated in (Table 5). This shows that the validity of the optimized wet decomposition method for the honey samples was the same for the acceptable range (80–120%) [29]. This result clearly supports the suitability and accuracy of our method for analyzing metals in honey.

**Table 5:** Percentage of recovery

Metals	number of replicates (n)	Concentration un-spiked Samples (µg/g)	The amount added (µg/g)	Concentration in spiked samples (µg/g)	Recovery (%)
Zn	3	1.98 ± 0.05	0.2	2.14 ± 0.02	80%±1
Cu	3	1.93 ± 0.04	0.2	2.15 ± 0.01	110%±0.5
Mn	3	1.01 ± 0.01	0.2	1.19 ± 0.04	90%±1.26
Cd	3	0.025 ± 0.001	0.1	0.12 ± 0.06	95%±2.5
Cr	3	0.28 ± 0.03	0.1	0.36 ± 0.02	80%±2

Concentration values are mean ± SD of triplicate readings of triplicate measurements

### Optimization of decomposition of honey samples

Nine different trials were tested for decomposition of the honey samples. These trials were developed with some adjustment of the procedure in the literature used to determine the metal content of honey samples by FAAS [6–27]. The optimized conditions and situation indicated under number 3 (Table 6) were used during the digestion.

**Table 6:** Optimization conditions

	Reagents used	Volume ratio (ml)	Temperature (^o^C)	Decomopstion time (hour)	Observations
1	HNO_3_:H_2_O_2_	5:3	200	3	Clear yellow
2	HNO_3_: H_2_O_2_	4:3	200	3	Yellow
3	HNO_3_:H_2_O_2_	3:1	150	2.5	Clear colorless
4	HNO_3_:H_2_O_2_	3:5	150	2	Yellow
5	HNO_3_:HClO_4_	5:1	250	3	Clear Colorless
6	HNO_3_:HClO_4_	5:2	250	3	Pale yellow
7	HNO_3_:HClO_4_	3:5	200	2	Yellow
8	HNO_3_:HClO_4_:H_2_O_2_	5:3:1	300	3.5	Colorless
9	HNO_3_:HClO_4_:H_2_O_2_	5:3:2	300	3.5	Clear yellow

The higher chemical composition, longer duration of complete decomopstion and observation of colors in some of the tested trials were the common drawbacks of other tested procedures.

### Concentration of heavy metals in honey samples

**Fig. 2:**
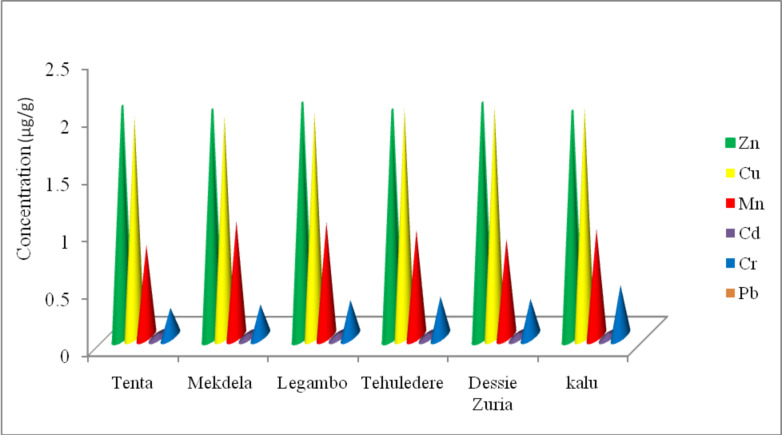
Mean ± SD of triplicate readings of triplicate measurements

The concentration of essential mineral of Zn, Cu and Mn become highest value than the other metals and the concentration Pb in all samples were below the detection limits. The result in (Table 2; Fig. 2), concentration of Zn is the highest (1.97 µg/g − 2.04 µg/g) followed by copper (1.92 µg/g – 2 µg/g), manganese (0.83 µg/g − 1.01 µg/g), chromium (0.25 µg/g − 0.45 µg/g), and cadmium (0.025 µg/g − 0.031 µg/g) whereas lead was below the detection limit all honey samples (Table 2). Statically, For Cu and Mn, no significant differences at the 95% confidence level (p ≥ 0.05) were observed the mean concentrations between all the six honey samples. But Zn, Cd and Cr differ significantly (p < 0.05) were observed in the six honey samples.

## Discussion

### Detection of concentration of heavy metals

The concentration of Mn, Cu, Zn, Cd, Cr, and Pb metals in the honey samples of six Woredas were determined by Flame Atomic Absorption Spectroscopy (Table 2). The concentration of essential mineral of Zn, Cu and Mn become highest value than the other metals and the concentration Pb in all samples were below the detection limits. The concentration of Zn is highest (1.97 µg/g − 2.04 µg/g) followed by copper (1.92 µg/g – 2 µg/g), manganese (0.83 µg/g − 1.01 µg/g), chromium (0.25 µg/g − 0.45 µg/g), and cadmium (0.025 µg/g − 0.031 µg/g) whereas lead was below the detection limit in all honey samples.

**Copper (Cu)** is essential for a variety of biochemical processes and is needed for enzymes in the body. It is also involved in the functioning of the nervous system, and in maintaining the balance of other useful trace metals in the body. Although copper homeostasis plays an important role in the prevention of copper toxicity, exposure to excessive levels of copper can result in a number of adverse health effects including liver and kidney damage, anemia, immunotoxicity, and developmental toxicity. Many of these effects are consistent with oxidative damage to membranes or macromolecules [30].

**Manganese (Mn)** is a trace mineral that is present in tiny amounts in the body. It is one of the most important nutrients for human health. The average human body contains about 12 mg of Mn. Manganese helps the body to form connective tissue, bones, blood-clotting factors and sex hormones. It also plays a role in fat and carbohydrate metabolism, calcium absorption and blood sugar regulation [30].

**Chromium (Cr)** is a trace element that humans require in trace amounts. It is found primarily in two forms: Trivalent (chromium III), which is biologically active and found in food and hexavalent (chromium VI), a toxic form that results from industrial pollution. Chromium produces significant increases in enzyme activity and serves an important function in carbohydrate metabolism, stimulation of fatty acid and cholesterol synthesis from acetate in the liver and improved sugar metabolism through the activation of insulin [37].

Statistically, Cu and Mn do not have significant differences at the 95% confidence level (p ≥ 0.05). However, Zn, Cd and Cr have statistically significant differences (p < 0.05) in the six honey samples. The mount of intake of above the presumable limits causes of Zn for gastrointestinal distress and diarrhea [30]. Copper for liver and kidney damage [4] and Mn for hypertension and irreversible neurological disorders [31]. Chromium required for maintenance of normal glucose metabolism, related to the function of insulin and Cr (III) complexes play a key role in carbohydrate and lipid metabolism [32]. The concentrations of Pb in all tested sampling sites were below the detection limit. All results indicated that the South Wollo Honey is below presumable limits means important, preferable and healthy. The sample analyzed of non-essential metal selected sampling sites were below maximum permissive limits set by the World Health Organization [32, 33]. Thus, South Wollo honeys did not cause a health risk to consumers instead of a problem related to Cd, Cr, and Pb.

The essential metal concentrations of Zn (1.97–2.04 µg/g), Cu (1.92–2.02 µg/g) and Mn (0.83–1.01 µg/g) in South Wollo Zone honey were higher than the values reported on Sidama Zone, Southern Nations by [33, 34], Debre Nazareth of Tigray by [5, 36], and East Wollega Zone of Oromia by [36], in Ethiopia also Turkey honey by [37] and kenya by [38, 39] (Table 3).

Cadmium is a cumulative toxic agent with half-life of several years and their burden of the body increases with age. Cadmium and solutions of its compounds are toxic, chronic exposure can cause irreversible damage to the lungs and eventually, death. Eating food or drinking water with high cadmium concentration irritates the stomach causing vomiting and diarrhea. It accumulates in the kidney and liver causing kidney dysfunctioning and liver failure, in addition to being a teratogenic and carcinogenic agent [27].

Zinc has numerous functions in the body and it is essential element for human health. At the same time, zinc function as a cofactor for many enzymes of the body. Excess intake of zinc in to the body through food, water or dietary supplements can affect health. If large doses of zinc by mouth even for a short time, stomach cramps, nausea and vomiting may occur. Ingesting high levels of zinc for several months may cause anemia, damage the pancreas and decrease levels of high – density lipoprotein (HDL) cholesterol [39].

### Comparison of the current study with other reported values

Comparing the results of this study with the results of the studies done so far are very essential to assess the accuracy and precision of the results found from this research. The obtained concentrations level of heavy metals in South Wollo of Cd (0.025–0.031 µg/g) was very low as compared to East Wollega by [33, 36]. But the concentration of Cd was consistent with Kenya honey and Debre Nazareth of Tigray as reported by [35, 36]. The concentrations of Zn (1.97–2.04 µg/g) in honey were lower than the values reported by [37] ranged from (1.92–4.22 µg/g) and [30] ranged from (2.03–6.8 µg/g). The concentration of Zn values higher than the values reported on Ethiopia by [13, 34] the value ranged (0.062–0.335 and by [36] the value ranged from (0.65–0.93 µg/g) [25], the mean value was 0.48 µg/g [37].

As can be seen from Table 2, the concentration of Copper ranged from (1.92–2.02 µg/g) in honey determined in this study was higher than reported by [5, 25, 32, 33, 36, 37], whereas, Lead was not detected in this study.

The concentrations of Cd as shown in Table 6was very low compared to the values obtained by [33], ranged from (0.152–0.201 µg/g) [37], ranged from (ND-0.69 µg/g) and [34] the mean value was 0.088 µg/g [22]. However, the concentrations of Cd were consistent with the values obtained in Ethiopia and Kenya honey which ranged from 0.02 to 0.03 µg/g and 0.01–0.05 µg/g as reported by [36] and by [5], respectively [18]. Honey from Ethiopia had a very low concentration of Mn (0.0693–0.815 µg/g) as reported by [32]. The detection of the contamination of honey with heavy metals (Cd, Cr, Mn, Pb, Cu, and Zn) as shown in Table 2 had all metal concentrations below the maximum permissive limit set by WHO [28]. This study showed that the honey produced in selected South Wollo Zone was suitable for human consumption.

## Conclusion

This paper analyzed the concentration of selected heavy metals from honey samples from six-selected Woredas of South Wollo Zone. The average concentration of heavy metals such as Zn, Cu, Mn, Cr, and Cd was 2 µg/g, 1.97 µg/g, 0.93 µg/g, 0.33 µg/g and 0.028 µg/g, respectively. The lead (Pb) was not detected in all honey samples. Therefore, the honey produced in selected Woredas has good quality for human consumption. The proximity to the industries, having different types of soil, using various fertilizers, and the diversity in practice of growing the plants may be led to some differences between regions. Heavy metals concentration in different areas depends on various variables, leading to their different concentrations in honey. To minimize the adverse effects, quality control of food products, monitoring the soils in agricultural lands and limiting fertilizers use are recommended. The present investigation of heavy metals in honey samples of South Wollo revealed that the area is less contaminated with heavy metals. The region has considerable area of natural forests and plantation crops. Hence, the honey samples collected in the selected areas has less heavy metal but are at the acceptable levels for human consumption.

## Data Availability

All data generated and analyzed are included within this research article.
